# Lower Geriatric Nutritional Risk Index (*GNRI*) Is Associated with Higher Risk of Fractures in Patients Undergoing Hemodialysis

**DOI:** 10.3390/nu13082847

**Published:** 2021-08-19

**Authors:** Maria Yoshida, Ayumu Nakashima, Shigehiro Doi, Kazuya Maeda, Naoki Ishiuchi, Takayuki Naito, Takao Masaki

**Affiliations:** 1 Department of Nephrology, Hiroshima University Hospital, 1-2-3 Kasumi, Minami-ku, Hiroshima 734-8551, Japan; mariayoshida@hiroshima-u.ac.jp (M.Y.); sdoi@hiroshima-u.ac.jp (S.D.); kazu_kazu0725@yahoo.co.jp (K.M.); naoki.ishiuchi@gmail.com (N.I.); 2 Department of Stem Cell Biology and Medicine, Graduate School of Biomedical & Health Sciences, Hiroshima University, 1-2-3 Kasumi, Minami-ku, Hiroshima 734-8553, Japan; 3 Ichiyokai Yokogawa Clinic, 2-7-9 Yokogawacho, Nishi-ku, Hiroshima 733-0011, Japan; t.naito@do.enjoy.ne.jp

**Keywords:** bone fracture, hemodialysis, malnutrition, geriatric nutritional risk index

## Abstract

Background: Although malnutrition and bone fracture are both major complications in patients undergoing hemodialysis, their association has not been clarified. The aim of our study was to clarify the association between the geriatric nutritional risk index (*GNRI*), an indicator of nutritional status, and the incidence of bone fractures in patients undergoing hemodialysis. Methods: We included 1342 registered patients undergoing hemodialysis and performed a post hoc analysis. We divided patients into the high *GNRI* group (≥92), considered to have a low risk of malnutrition, and the low *GNRI* group (<92), considered to have a high risk of malnutrition. Fracture-free survival in the low and high *GNRI* groups was evaluated by the Kaplan–Meier method. Cox proportional hazards models were used to identify the risk factors for fractures requiring hospitalization. All results were stratified by sex. Results: New bone fractures developed in 108 (8.0%) patients in 5 years of follow-up. Bone fractures occurred more frequently in the low *GNRI* group compared with the high *GNRI* group (HR: 3.51, 95% CI: 1.91–6.42, *p* < 0.01 in males; HR: 2.47, 95% CI: 1.52–4.03, *p* < 0.01 in females). A low *GNRI* was significantly associated with an increased incidence of bone fractures, even after adjustment for covariates. However, the serum levels of calcium, phosphate, parathyroid hormone, and alkaline phosphatase were not associated with the incidence of bone fractures. Conclusions: A low *GNRI* is an independent risk factor for bone fractures in patients undergoing hemodialysis. Early intervention for the low *GNRI* group may be important in preventing the occurrence of fractures.

## 1. Introduction

In patients undergoing dialysis, fracture is a serious complication that results in a decreased quality of life, increased economic burden, and increased mortality rate [1,2]. Fracture is highly prevalent in patients undergoing hemodialysis compared with the non-hemodialysis population [3]. To improve the quality of life and prognosis of patients undergoing hemodialysis, there is a need for the early identification of those at high risk of fracture.

Patients undergoing hemodialysis also frequently experience malnutrition. Previous surveys have shown that malnutrition affects about 40% of patients undergoing hemodialysis [4]. A study that used the modified subjective global assessment showed that 18% of dialysis patients have moderate to severe malnutrition [5]. As a single indicator of malnutrition is not sufficient clinically, the following indicators have been used in combination to detect malnutrition in patients undergoing hemodialysis: the subjective global assessment, malnutrition-inflammation score, normalized protein catabolic rate, creatinine (Cr) index, and geriatric nutritional risk index (*GNRI*). The *GNRI*, evaluated based on serum albumin level and body mass index (BMI), was originally developed as a predictor of the risks of morbidity and mortality in hospitalized older adult patients [6]. For patients undergoing hemodialysis, the *GNRI* is often used as a useful and simple indicator of malnutrition [7] and as a predictor of cardiovascular diseases and mortality [8,9].

While both malnutrition and fracture are major complications that develop in patients undergoing hemodialysis, little information is available on the association between malnutrition and fracture. A previous study showed that low serum albumin is associated with new fractures [10]. However, the association between a low BMI and fractures remains controversial [3,10,11]. We hypothesized that malnutrition is associated with fracture, and that the *GNRI*, as an indicator of malnutrition, can predict the risk of fractures. We herein compared the 5-year incidence of fractures requiring hospitalization between the high and low *GNRI* groups in a population of patients undergoing dialysis. Our findings provide insights into the prevention of fractures in patients undergoing hemodialysis.

## 2. Materials and Methods

### 2.1. Study Population

**Figure 1:**
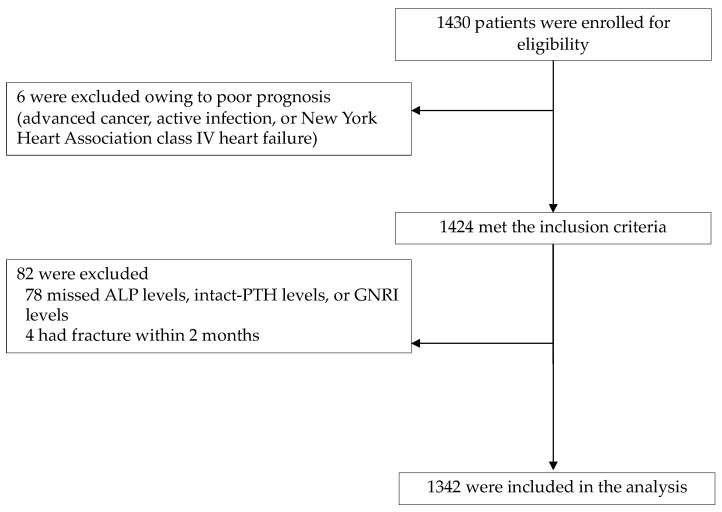
Flow chart of the cohort. Abbreviations: CRP, C-reactive protein; *GNRI*, geriatric nutritional risk index; intact-PTH, intact parathyroid hormone; ALP, alkaline phosphatase.

This study was performed at 13 dialysis units in Hiroshima, Japan. This was a retrospective, follow-up study and a post hoc analysis of baseline data arising from a study originally aimed at investigating a biomarker for the detection of mortality in patients undergoing hemodialysis [12]. In the original study, patient recruitment took place between December 2011 and November 2012. The end of follow-up was November 2017. Patients treated with maintenance hemodialysis therapy with three sessions per week and aged 20 years or older were enrolled. Overall, 1430 outpatients were initially enrolled in the study. Patients were excluded if they were treated with other modalities of dialysis therapy, had undergone more or less than three hemodialysis sessions per week, and had a poor prognosis because of advanced cancer, active infection, or New York Heart Association class IV heart failure. A total of 1424 patients were included in the present study and they provided written informed consent. Patients were excluded from the present analysis if there were missing data regarding *GNRI* levels, alkaline phosphatase (ALP), or parathyroid hormone (PTH) levels or if they were suffered from fractures requiring hospitalization within 2 months. The remaining 1342 patients were included in the present analysis. Figure 1 shows flow diagrams of this study. We defined smokers as current smokers and ever smokers. The Ethics Committee of our hospital approved the study protocol (approval number E-2141, registered 6 August 2020). This study was conducted in accordance with the principles contained within the Declaration of Helsinki.

### 2.2. Data Collection

Pre-hemodialysis blood samples were collected at the first dialysis session of the week. Samples were examined at standardized laboratories that had contracts with each hemodialysis clinic. Clinical data including hemodialysis conditions, number of days of hospitalization during the observation period, and medications were collected from the medical records. Fracture events were defined as any fracture requiring hospitalization.

### 2.3. Calculation of the GNRI

The *GNRI* was developed as a screening tool for the risks of morbidity and mortality in hospitalized older adult patients [5]. In accordance with a previous study [6], the *GNRI* was calculated using the following formula:

GNRI = [14.89 × serum albumin (g/dL)] + [41.7 × (actual body weight/ideal body weight)]

ideal body weight (kg) = [height (m)]^2^ × 22 (kg/m^2^)

The body weight or ideal body weight was set to 1 when the patient’s body weight was greater than the ideal body weight. Several previous studies have shown that a *GNRI* cut-off value of 92 identifies malnourished patients, particularly in patients undergoing hemodialysis [7,8,13]. Therefore, we used the *GNRI* cut-off value of 92 to divide the patients into the high *GNRI* group (≥92) considered to have a low risk of malnutrition, and the low *GNRI* group (<92) considered to have a high risk of malnutrition. 

### 2.4. Statistical Analysis

The Mann–Whitney *U* test or chi-squared test was used to compare each variable between the high and low *GNRI* groups. All variables were expressed as the mean ± standard deviation or median and interquartile range (25th to 75th percentile), unless otherwise indicated. All results were stratified by sex because the rate of hip fracture is higher among females than males in both the dialysis population and the general population. Statistical significance was set at *p* < 0.05. Logistic regression approaches were used to assess the determinants of an existing low *GNRI* (<92). Categories for age, dialysis vintage, and levels of hemoglobin, C-reactive protein, total cholesterol, Cr, calcium (Ca), phosphate (Pi), ALP, and intact-PTH were calculated in accordance with the median value of the group.

Fracture-free survival was plotted by the Kaplan–Meier estimation method. The log-rank test was used to analyze significance. Cox proportional hazards models were used to identify the risk factors for fractures, leading to hazard ratios (HRs) and 95% confidence intervals (CIs). The selected covariates were the *GNRI* and factors that have been identified as predictors of fractures or secondary hyperparathyroidism in previous studies [14,15,16]. Categories for age, dialysis vintage, levels of hemoglobin, C-reactive protein, total cholesterol, Cr, Ca, Pi, ALP, intact-PTH, and *GNRI* were calculated in accordance with the median value of the group. After evaluating the crude HR, we adjusted the HRs for the covariates. All analyses were carried out with JMP^®^ 14.2.0 (SAS Institute Inc., Cary, NC, USA).

## 3. Results

### 3.1. Patient Characteristics

Baseline characteristics stratified by the *GNRI* are shown in Table 1. Compared with the high *GNRI* group (≥92), both male and female patients with a low *GNRI* (<92) were older, had a lower BMI, lower dry weight, lower levels of albumin, Cr, Ca, Pi, and intact-PTH, a higher ALP level, and a lower proportion of patients with prescriptions for Pi-binders. Supplementary Table S1 describes the baseline characteristics stratified by the *GNRI* of all patients.

### 3.2. Factors Associated with a Low GNRI (<92)

Multiple regression analysis was performed to identify factors associated with a low *GNRI* (<92). As shown in Table 2, for both sexes, the factors significantly associated with a low *GNRI* (<92) were older age, lower Cr level, and lower Ca level. In addition, in males, a longer dialysis vintage, lower hemoglobin level, and higher C-reactive protein level were related to a low *GNRI* (<92). In females, a higher prevalence of diabetes mellites, use of statins, use of vitamin D receptor activators, and use of erythropoiesis-stimulating agents were associated with a low *GNRI* (<92). The findings remained even after adjusting for sex (Supplementary Table S2).

### 3.3. Association between the GNRI and Risk of Bone Fractures

**Figure 2:**
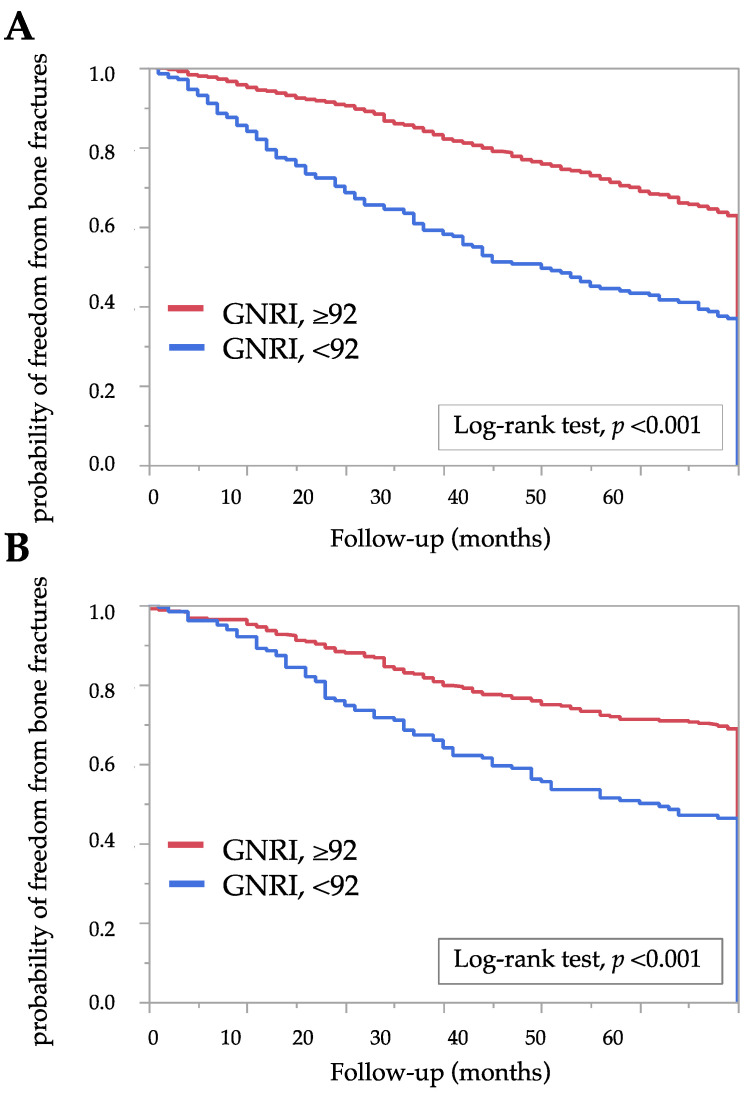
Kaplan–Meier estimates of the probability of freedom from bone fractures according to two *GNRI* groups: (**A**) male and (**B**) female. The log-rank test was used in analysis. A two-tailed *p* < 0.05 was considered statistically significant. Abbreviations: *GNRI*, geriatric nutritional risk index.

New bone fractures requiring hospitalization occurred in in 108 patients (8.0%) during 5 years of follow-up. We followed all patients for 5 years, except in the case of events such as hospital transfer (*n* = 196), kidney transplant (*n* = 19), transition to four sessions per week hemodialysis (*n* = 21), transition to home hemodialysis (*n* = 1), and death (*n* = 350). Therefore, the median value for follow-up time was 60 (28–60) months. Figure 2 shows the Kaplan–Meier curves in the two *GNRI* groups for the probability of freedom from bone fractures. The Kaplan–Meier analysis showed that bone fractures occurred more frequently in the low *GNRI* group compared with the high *GNRI* group (log-rank test, *p* < 0.01). To evaluate the association between covariates and the incidence of bone fractures in 60 months, we estimated the HRs for bone fractures using Cox proportional hazard ratio models. The incidence of bone fractures was significantly correlated with a low *GNRI* (<92) (HR: 3.51, 95% CI: 1.91–6.42 in males; HR: 2.47, 95% CI: 1.52–4.03 in females). Table 3 shows the HRs after adjusting for covariates. Even after adjustment, a low *GNRI* (<92) was significantly associated with increased risk of bone fractures (HR: 2.93, 95% CI: 1.54–5.59 in males; HR: 2.05, 95% CI: 1.20–3.51 in females). In contrast, the levels of ALP, Ca, Pi, and intact-PTH were not associated with an increased risk of fractures. These associations persist even when using the *GNRI* as a continuous variable. The *GNRI* was significantly associated with fracture (HR: 3.21, 95% CI: 2.20–4.70, *p* < 0.01) in the analysis of all patients (combining data for males and females). The association was retained even after adjusting for other variables, including sex (Supplementary Figure S1 and Table S3).

## 4. Discussion

This study was performed to demonstrate an association between the incidence of fractures and the *GNRI*, a parameter used to indicate the nutritional status in patients undergoing hemodialysis. As fracture is an important complication in patients undergoing hemodialysis, fracture prevention treatments, such as chronic kidney disease-mineral bone disorder (CKD-MBD) management, have been used [17]. Our results showed that a low *GNRI* (<92) was significantly associated with an increased risk of bone fractures. Moreover, we confirmed that malnutrition was more significantly associated with the incidence of fractures than other predictors reported in previous studies [3,10,18,19,20]. These findings indicate that the management of malnutrition, in addition to CKD-MBD management, might help to prevent fractures.

The following variables have previously been reported as predictors of fracture risk in patients undergoing dialysis: older age, female sex [3,10,18], extremely high PTH values [18], long-term dialysis [3,20], higher values of bone-specific ALP [20], and diabetes mellitus [19]. However, relatively little is known about whether nutritional indicators predict the incidence of fractures. A low albumin level is reportedly one of many factors associated with fractures [18], while an association between a low BMI and fractures remains controversial. One previous study showed that a low BMI is significantly associated with fractures [18], although other studies found no such association [3,11].

Although previous studies have showed that fractures are associated with several factors, such as duration of dialysis [3,19], diabetes mellitus [19], and PTH [18] and ALP values [20], the present study showed that the *GNRI* is more strongly associated with fractures than these predictors. The discrepancy between our study and the previous studies might be derived from the differences in patient characteristics. The mean age of Japanese patients in our study was older than that of the patients included in previous studies. In addition, our patients took lanthanum carbonate (27%) and cinacalcet (20%). We speculate that the intake of these drugs may have caused the lower PTH value observed in our study compared with previous studies. Racial disparities or differences in dialysis prescriptions among countries may also affect the incidence of fractures. These differences may reduce the impact of the clinical factors reported previously. Several studies have reported that serum calcium and serum phosphorus levels are not associated with the incidence of fractures [4,8]. However, we believe that serum phosphate levels reflect malnutrition because phosphate dietary content is closely associated with protein content. Therefore, we used these variables in the analyses in the present study. 

Patients with CKD with malnutrition frequently have protein energy wasting (PEW). PEW is characterized by the state of decreased body stores of protein and energy fuels, and is associated with uremic toxins, hypercatabolism, oxidative stress, and inflammation [21], which may lead to loss of bone strength. Based on the abovementioned information, we consider that the low *GNRI* group would probably have PEW. An association between the *GNRI* and inflammation has been reported [22,23]. Overall, we suggest that a low *GNRI* may detect disorders linked to decreased bone strength through PEW in patients undergoing hemodialysis.

Muscle weakness is one of the main causes of falls, and patients with CKD develop impaired balance and muscle weakness due to inactivity and myopathy, independent of alterations in bone mineral density [24]. Therefore, a low *GNRI* is thought to contribute to the occurrence of fractures via its association with muscle weakness as well as bone strength. The modified creatinine (Cr) index reflects skeletal muscle mass in patients on hemodialysis. A previous study has shown that the modified Cr index score is associated with an increased risk of bone fractures in both men and women on hemodialysis [25]. The Cr index and *GNRI* show similar clinical associations with the risk of mortality in hemodialysis patients [26]. These findings suggest that malnutrition is associated with fracture via muscle weakness and loss of bone strength.

The present study has some limitations. We have no information on the following known risk factors for bone fractures [18]: previous fracture history, family history of hip bone fracture, use of glucocorticoids, use of psychoactive medications [1], current alcohol consumption, and rheumatoid arthritis. We also did not measure bone mineral density, ALP isozymes, activities of daily living, muscle strength index data, such as the timed-up-and-go test or one-leg standing time, and frailty index data, such as the frailty phenotype or frailty index. Besides, bone fracture location and etiology were not investigated. In addition, we used raw data and did not convert between different laboratories. Despite these limitations, our study is unique in that we found that the *GNRI* was a predictor of fractures independent of the PTH or ALP values, which are the clinical markers of bone turnover. Our findings suggest that it is more important to manage nutrition than CKD-MBD to prevent fractures. Improved malnutrition may help to prevent loss of muscle mass and strength or to reduce inflammation, which may lead to a decrease in the incidence of fractures. We believe that our findings give clinically important information about the prevention of fractures in patients undergoing hemodialysis by using not only PTH management, but also malnutrition management.

In conclusion, the present study demonstrates for the first time that the *GNRI*, a simple surrogate for nutritional status, is useful in discriminating the risk of fractures in patients undergoing hemodialysis. The provision of early intervention in the low *GNRI* group may help prevent fractures. Further studies are needed to better understand the effect of malnutrition on the development of fractures in patients undergoing hemodialysis.

## Figures and Tables

**Table 1:** Clinical characteristics by *GNRI* of (**A**) males and (**B**) females.

(**A**)			
**Variables**	***GNRI***		***p* Value**
	**≥92 (*n* = 631)**	**<92 (*n* = 202)**	
Age, years	63.4 ± 12.3	72.3 ± 11.1	<0.001
Dialysis vintage, months	62 (26–124)	67 (29–133)	0.556
Diabetes mellitus, n (%)	251 (40.1)	76 (37.8)	0.564
Smokers (ever, current), n (%)	427 (67.8)	143 (71.1)	0.371
Dry weight, kg	61.7 ± 10.0	52.6 ± 9.4	<0.001
Hemoglobin level, g/dL	11.0 ± 1.1	10.5 ± 1.2	<0.001
CRP level, mg/L	0.9 (0.5–2.8)	2.7 (0.8–7.8)	<0.001
Total cholesterol level, mg/dL	151.5 ± 30.0	144.0 ± 31.8	<0.001
Cr level, mg/dL	11.6 ± 2.9	9.4 ± 2.6	<0.001
Ca level, mg/dL	9.0 ± 0.7	8.7 ± 0.8	<0.001
Pi level, mg/dL	5.2 ± 1.2	4.9 ± 1.4	<0.001
ALP level, U/L	217 (171–274)	242 (187–292)	0.004
Intact-PTH level, pg/mL	122 (67–188)	101 (47–153)	0.002
Use of statins, n (%)	110 (17.4)	26 (12.9)	0.127
Use of VDRAs, n (%)	388 (61.5)	113 (56.0)	0.161
Use of Pi-binders, n (%)	560 (82.3)	146 (72.3)	0.002
Use of ESAs, n (%)	560 (88.8)	191 (90.2)	0.016
(**B**)			
**Variables**	***GNRI***		***p* Value**
	**≥92 (*n* = 329)**	**<92 (*n* = 180)**	
Age, years	64.2 ± 11.3	70.8 ± 12.5	<0.001
Dialysis vintage, months	96 (33–175)	71 (31–133)	0.022
Diabetes mellitus, n (%)	112 (34.6)	43 (24.6)	0.021
Smokers (ever, current), n (%)	66 (20.1)	33 (18.4)	0.647
Dry weight, kg	51.8 ± 11.0	42.9 ± 8.2	<0.001
Hemoglobin level, g/dL	10.7 ± 1.1	10.7 ± 1.3	0.763
CRP level, mg/L	0.7 (0.5–1.9)	0.7 (0.5–2.9)	0.261
Total cholesterol level, mg/dL	172.0 ± 31.1	167.0 ± 36.0	0.042
Cr level, mg/dL	10.2 ± 2.2	8.6 ± 2.0	<0.001
Ca level, mg/dL	9.2 ± 0.7	8.9 ± 0.8	<0.001
Pi level, mg/dL	5.4 ± 1.3	5.2 ± 1.4	0.045
ALP level, U/L	239 (183–313)	273 (191–336)	0.036
Intact-PTH level, pg/mL	124 (69–195)	120 (55–180)	0.106
Use of statins, n (%)	96 (29.2)	30 (16.7)	0.002
Use of VDRAs, n (%)	199 (60.7)	116 (64.4)	0.402
Use of Pi-binders, n (%)	285 (86.9)	1313 (74.0)	<0.001
Use of ESAs, n (%)	301 (91.5)	165 (91.7)	0.945

Data are presented as mean ± standard deviation or median (interquartile range) for continuous variables. Differences between groups were analyzed using the Mann–Whitney U test or chi-squared test. Abbreviations: *GNRI*, geriatric nutritional risk index; CRP, C-reactive protein; Cr, creatinine; Ca, calcium: Pi, phosphate; ALP, alkaline phosphatase; intact-PTH, intact parathyroid hormone; VDRAs, vitamin D receptor activators; ESAs, erythropoiesis-stimulating agents.

**Table 2:** Odds ratios and 95% CI for factors predicting a *GNRI* of <92 in (**A**) males and (**B**) females.

(**A**)		
**Variable**	**Odds Ratio**	***p* Value **
Intercept		<0.001
Age, ≥67 years	2.02 (1.37–2.98)	<0.001
Dialysis vintage, ≥73 months	1.87 (1.25–2.82)	0.003
Diabetes mellitus, presence	0.69 (0.47–1.03)	0.070
Smokers	1.30 (0.87–1.94)	0.200
Hemoglobin level, ≥10.9 g/dL	0.58 (0.40–0.83)	0.003
CRP level, ≥1.0 mg/L	2.28 (1.56–3.34)	<0.001
Total cholesterol level, ≥156 mg/dL	0.70 (0.48–1.02)	0.065
Cr level, ≥10.5 mg/dL	0.29 (0.19–0.45)	<0.001
Ca level, ≥9.1 mg/dL	0.59 (0.40–0.88)	0.010
Pi level, ≥5.2 mg/dL	0.96 (0.66–1.41)	0.847
ALP level, ≥230 U/L	1.32 (0.91–1.92)	0.144
Intact-PTH level, ≥118 pg/mL	0.68 (0.46–1.01)	0.055
Use of statins, presence	0.81 (0.48–1.37)	0.438
Use of VDRAs, presence	0.83 (0.57–1.21)	0.334
Use of Pi-binders, presence	0.85 (0.54–1.35)	0.490
Use of ESAs, presence	2.14 (1.02–4.45)	0.044
(**B**)		
**Variables**	**Odds Ratios**	***p* Value **
Intercept		<0.001
Age, ≥67 years	2.08 (1.32–3.28)	0.002
Dialysis vintage, ≥73 months	0.81 (0.51–1.27)	0.350
Diabetes mellitus, presence	0.48 (0.30–0.77)	0.003
Smokers	0.92 (0.53–1.60)	0.760
Hemoglobin level, ≥10.9 g/dL	1.30 (0.85–1.99)	0.230
CRP level, ≥1.0 mg/L	1.01 (0.66–1.56)	0.947
Total cholesterol level, ≥156 mg/dL	0.65 (0.41–1.00)	0.050
Cr level, ≥10.5 mg/dL	0.33 (0.18–0.55)	<0.001
Ca level, ≥9.1 mg/dL	0.45 (0.29–0.70)	<0.001
Pi level, ≥5.2 mg/dL	0.88 (0.56–1.36)	0.557
ALP level, ≥230 U/L	1.42 (0.91–2.24)	0.121
Intact-PTH level, ≥118 pg/mL	0.83 (0.53–1.30)	0.428
Use of statins, presence	0.37 (0.21–0.65)	<0.001
Use of VDRAs, presence	1.75 (1.10–2.78)	0.017
Use of Pi-binders, presence	0.61 (0.35–1.05)	0.073
Use of ESAs, presence	0.39 (0.18–0.85)	0.018

Table 2 (**A**): The adjusted R^2^ of the model was 0.178. Table 2 (**B**): The adjusted R^2^ of the model was 0.180. Categories for age, dialysis vintage, and levels of hemoglobin, CRP, total cholesterol, Cr, Ca, Pi, ALP, and intact-PTH were calculated in accordance with the median value of the group. Abbreviations: CRP, C-reactive protein; Cr, creatinine; Ca, calcium: Pi, phosphate; ALP, alkaline phosphatase; intact-PTH, intact parathyroid hormone; VDRAs, vitamin D receptor activators; ESAs, erythropoiesis-stimulating agents.

**Table 3:** Hazard ratios for bone fractures in (**A**) males and (**B**) females.

(**A**)		
**Univariate**	**HR (95% CI)**	***p* Value **
*GNRI*, <92	3.51 (1.91–6.42)	<0.001
**Covariate**	**HR (95% CI)**	***p* Value **
Age, ≥67 years	1.99 (1.04–3.81)	0.038
Dialysis vintage, ≥73 months	0.77 (0.39–1.50)	0.439
Smokers (ever, current), presence	0.79 (0.43–1.48)	0.470
CRP level, ≥1.0 mg/L	0.93 (0.50–1.72)	0.815
Ca level, ≥9.1 mg/dL	0.82 (0.42–1.65)	0.584
Pi level, ≥5.2 mg/dL	1.36 (0.73–2.55)	0.337
ALP level, ≥230 U/L	1.54 (0.82–2.89)	0.179
Intact-PTH level, ≥118 pg/mL	0.72 (0.37–1.38)	0.323
Use of VDRAs, presence	1.32 (0.70–2.51)	0.390
Use of Pi-binders, presence	0.62 (0.31–1.25)	0.181
*GNRI*, <92	2.94 (1.54–5.59)	0.001
(**B**)		
**Univariate**	**HR (95% CI)**	***p* Value **
*GNRI*, <92	2.47 (1.52–4.03)	<0.001
**Covariate**	**HR (95% CI)**	***p* Value **
Age, ≥67 years	1.76 (1.02–3.04)	0.042
Dialysis vintage, ≥73 months	0.93 (0.55–1.55)	0.775
Smokers (ever, current), presence	0.65 (0.32–1.36)	0.255
CRP level, ≥1.0 mg/L	1.03 (0.61–1.72)	0.917
Ca level, ≥9.1 mg/dL	0.97 (0.56–1.66)	0.908
Pi level, ≥5.2 mg/dL	1.36 (0.80–2.30)	0.254
ALP level, ≥230 U/L	0.98 (0.57–1.67)	0.943
Intact-PTH level, ≥118 pg/mL	0.88 (0.52–1.47)	0.616
Use of VDRAs, presence	1.12 (0.66–1.90)	0.666
Use of Pi-binders, presence	0.58 (0.32–1.05)	0.072
*GNRI*, <92	2.05 (1.20–3.51)	<0.001

The presented HRs are for bone fractures. Categories for age, dialysis vintage, levels of CRP, Ca, Pi, ALP, and intact-PTH, as well as *GNRI* were calculated in accordance with the median value of the group. Abbreviations: CRP, C-reactive protein; Ca, calcium: Pi, phosphate; ALP, alkaline phosphatase; intact-PTH, intact parathyroid hormone; VDRAs, vitamin D receptor activators; *GNRI*, geriatric nutritional risk index.

## Data Availability

The data that support the findings of this study are available from the corresponding author upon reasonable request.

## Supplementary Materials

The following are available online at https://www.mdpi.com/article/10.3390/nu13082847/s1, Table S1: Clinical characteristics by *GNRI* of all patients, Table S2: Odds ratios and 95% CI for factors predicting a *GNRI* of <92, Figure S1: Kaplan-Meier estimates of the probability of freedom from bone fractures according to two *GNRI* groups. The log-rank test was used in analysis, Table S3: Hazard ratios for bone fractures.
