# Supplementary material for: Lower Geriatric Nutritional Risk Index (GNRI) Is Associated with Higher Risk of Fractures in Patients Undergoing Hemodialysis
Source: Nutrients. 2021 Aug 19;13(8):2847. doi: 10.3390/nu13082847 (PMC8400213; doi:10.3390/nu13082847)
Supplement: Supplementary file 1 [file nutrients-13-02847-s001.zip › nutrients-1321253-supplementary.pdf]

nutrients MDPI

Table S1. Clinical characteristics by *GNRI* of all patients.

Variables	*GNRI*		*p* Value
	≥92 (n = 960)	<92 (n = 382)	
Age, years	63.7 ± 12.0	71.6 ± 11.8	<0.001
Male, n (%)	631 (65.7)	202 (52.9)	<0.001
Dialysis vintage, months	73 (28–143)	68 (30–132)	0.604
Diabetes mellitus, n (%)	363 (38.2)	119 (31.6)	0.025
Smokers (ever, current), n (%)	493 (51.4)	176 (46.3)	0.090
Dry weight, kg	58.3 ± 11.4	48.0 ± 10.1	<0.001
Hemoglobin level, g/dL	10.9 ± 1.1	10.6 ± 1.2	<0.001
CRP level, mg/L	0.8 (0.5–2.3)	1.4 (0.5–5.6)	<0.001
Total cholesterol level, mg/dL	158.5 ± 31.9	154.8 ± 35.7	0.017
Cr level, mg/dL	11.1 ± 2.8	9.0 ± 2.3	<0.001
Ca level, mg/dL	9.1 ± 0.7	8.8 ± 0.8	0.003
Pi level, mg/dL	5.3 ± 1.3	5.0 ± 1.4	<0.001
ALP level, U/L	223 (175–287)	254 (188–318)	0.001
Intact-PTH level, pg/mL	122.2 (68–189)	113 (51–169)	0.002
Use of statins, n (%)	206 (21.4)	56 (14.7)	0.005
Use of VDRAs, n (%)	587 (61.2)	229 (60.0)	0.670
Use of Pi-binders, n (%)	804 (83.8)	269 (73.0)	<0.001
Use of ESAs, n (%)	861 (89.7)	356 (93.1)	0.046

Data are presented as mean ± standard deviation or median (interquartile range) for continuous variables. Differences between groups were analyzed using the Mann-Whitney U test or chi-squared test. Abbreviations: *GNRI*, geriatric nutritional risk index; CRP, C-reactive protein; Cr, creatinine; Ca, calcium: Pi, phosphate; ALP, alkaline phosphatase; intact-PTH, intact parathyroid hormone; VDRAs, vitamin D receptor activators; ESAs, erythropoiesis-stimulating agents.

Table S2. Odds ratios and 95% CI for factors predicting a *GNRI* of <92.

Variable	Odds ratio	*p* Value
Intercept		<0.001
Age, ≥67 years	1.98 (1.49–2.64)	<0.001
Sex, male	0.59 (0.42–0.83)	0.002
Dialysis vintage, ≥73 months	1.43 (1.00–1.81)	0.048
Diabetes mellitus, presence	0.61 (0.45–0.82)	0.001
Smokers (ever, current), presence	1.16 (0.85–1.58)	0.358
Hemoglobin level, ≥10.9 g/dL	0.81 (0.62–1.06)	0.124
CRP level, ≥1.0 mg/L	1.59 (1.21–2.09)	<0.001
Total cholesterol level, ≥156 mg/dL	0.68 (0.51–0.90)	0.007
Cr level, ≥10.5 mg/dL	0.32 (0.23–0.45)	<0.001
Ca level, ≥9.1 mg/dL	0.55 (0.41–0.73)	<0.001
Pi level, ≥5.2 mg/dL	0.92 (0.69–1.21)	0.534
ALP level, ≥230 U/L	1.37 (1.04–1.81)	0.027
Intact-PTH level, ≥118 pg/mL	0.76 (0.57–1.01)	0.061
Use of statins, presence	0.56 (0.39–0.82)	0.003
Use of VDRAs, presence	1.06 (0.80–1.40)	0.680
Use of Pi-binders, presence	0.81 (0.58–1.13)	0.213
Use of ESAs, presence	1.17 (0.71–1.93)	0.536

The adjusted R^2 of the model was 0.157. Categories for age, dialysis vintage, and levels of hemoglobin, CRP, total cholesterol, Cr, Ca, Pi, ALP, and intact-PTH were calculated in accordance with the median value of the group.

Abbreviations: CRP, C-reactive protein; Cr, creatinine; Ca, calcium: Pi, phosphate; ALP, alkaline phosphatase; intact-PTH, intact parathyroid hormone; VDRAs, vitamin D receptor activators; ESAs, erythropoiesis-stimulating agents.

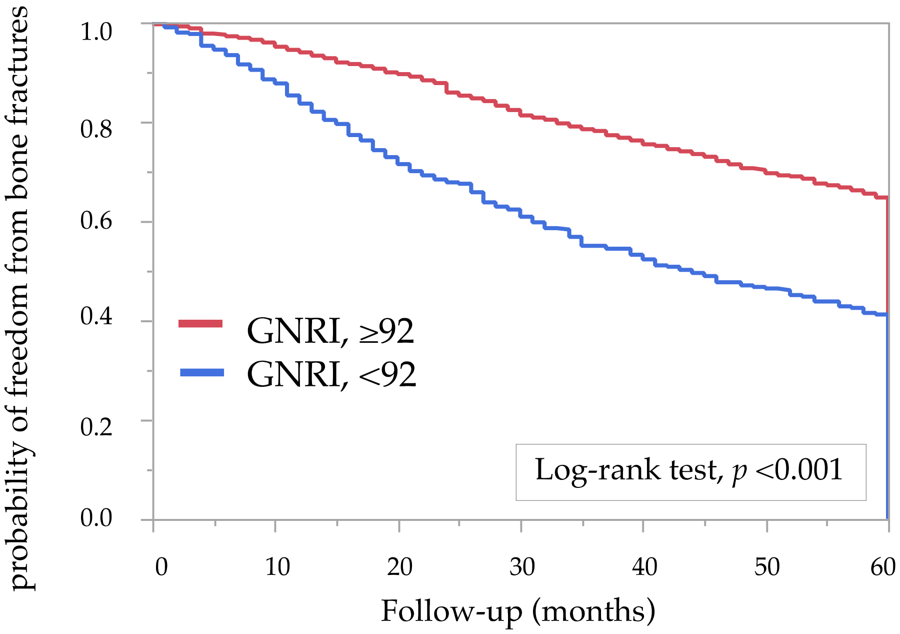

Figure S1. Kaplan-Meier estimates of the probability of freedom from bone fractures according to two *GNRI* groups. The log-rank test was used in analysis. A two-tailed $p < 0.05$ was considered statistically significant. Abbreviations: *GNRI*, geriatric nutritional risk index.

Table S3. Hazard ratios for bone fractures.

**Univariate**	**HR (95% CI)**	***p* Value**
GNRI, <92	3.21 (2.20–4.70)	<0.001
**Covariate**	**HR (95% CI)**	***p* Value**
Age, ≥67 years	1.83 (1.21–2.77)	0.004
Sex, male	0.52 (0.33–0.82)	0.005
Dialysis vintage, ≥73 months	0.88 (0.58–1.32)	0.536
Smokers (ever, current), presence	0.73 (0.46–1.15)	0.176
CRP level, ≥1.0 mg/L	1.00 (0.68–1.48)	0.991
Ca level, ≥9.1 mg/dL	0.92 (0.60–1.40)	0.692
Pi level, ≥5.2 mg/dL	1.37 (0.92–2.04)	0.122
ALP level, ≥230 U/L	1.17 (0.78–1.77)	0.445
Intact-PTH level, ≥118 pg/mL	0.81 (0.54–1.21)	0.309
Use of VDRAs, presence	1.18 (0.79–1.76)	0.425
Use of Pi-binders, presence	0.59 (0.38–0.92)	0.020
GNRI, <92	2.33 (1.54–3.52)	<0.001

The presented HRs are for bone fractures. Categories for age, dialysis vintage, levels of CRP, Ca, Pi, ALP, and intact-PTH, and *GNRI* were calculated in accordance with the median value of the group. Abbreviations: CRP, C-reactive protein; Ca, calcium: Pi, phosphate; ALP, alkaline phosphatase; intact-PTH, intact parathyroid hormone; VDRAs, vitamin D receptor activators; *GNRI*, geriatric nutritional risk index.
